# The adverse maternal and perinatal outcomes of adolescent pregnancy: a cross sectional study in Hebei, China

**DOI:** 10.1186/s12884-020-03022-7

**Published:** 2020-06-01

**Authors:** Ting Zhang, Huien Wang, Xinling Wang, Yue Yang, Yingkui Zhang, Zengjun Tang, Li Wang

**Affiliations:** 1 Department of Obstetrics and Gynecology, Hebei General Hospital, Hebei Medical University, Shijiazhuang, Hebei China; 2 Graduate School, Hebei North University, Zhangjiakou, Hebei China; 3 Department of Thoracic Surgery, Hebei General Hospital, Shijiazhuang, Hebei China; 4 Hebei Women and Children’s Health Center, Shijiazhuang, China

**Keywords:** Adolescent, Pregnancy, Maternal outcomes, Perinatal outcomes

## Abstract

**Background:**

The adverse pregnancy outcomes caused by teenage pregnancy are major public health problems with significant social impact. While China is the most populous country in the world, and 8.5% of the women aged 10–50 years are adolescent women, we aimed to analyze the adverse maternal and perinatal outcomes of the adolescent pregnancy in Hebei Province, China.

**Methods:**

There were 238,598 singleton pregnant women aged 10–34 years from January 1, 2013 to December 31, 2017 in the database of Hebei Province Maternal Near Miss Surveillance System (HBMNMSS). The 238,598 pregnant women were divided into two groups: adolescent group (aged 10–19 years) and adult group (aged 20–34 years). The adolescent group was divided into two subgroups (aged 10–17 years, aged 18–19 years), the adult group was divided into two subgroups (aged 20–24 years, aged 25–34 years). We compared the risk of adverse pregnancy outcomes using univariate and multivariate logistic regression. We also made a stratified analysis of nulliparous and multiparous adolescent pregnancy.

**Results:**

Compared with women aged 20–34 years, women aged 10–19 years had lower risk of cesarean delivery [adjusted risk ratio (aRR): 0.75, 95% confidence interval (CI): 0.70–0.80], gestational diabetes mellitus (GDM) (aRR: 0.55, 95%CI: 0.41–0.73). Women aged 10–19 years had higher risk of preterm delivery (aRR: 1.76, 95%CI: 1.54–2.01), small for gestational age (SGA) (aRR: 1.19, 95%CI: 1.08–1.30), stillbirth (aRR: 2.58, 95%CI: 1.83–3.62), neonatal death (aRR: 2.63, 95%CI: 1.60–4.32). The adolescent women aged 10–17 years had significantly higher risk of stillbirth (aRR: 5.69, 95%CI: 3.36–9.65) and neonatal death (aRR: 7.57, 95%CI: 3.74–15.33) compared with the women aged 25–34 years. Younger adults (20–24 years) also had higher risks of preterm delivery (aRR: 1.26, 95%CI: 1.20–1.32), stillbirth (aRR: 1.45, 95%CI: 1.23–1.72), and neonatal death (aRR: 1.51, 95%CI: 1.21–1.90) compared with women aged 25–34 years. The structural equation model showed that preterm delivery and cesarean delivery had an indirect effect on neonatal death in adolescent pregnancy.

**Conclusions:**

The adolescent pregnancy was related to adverse perinatal (fetal and neonatal) outcomes, such as preterm delivery, stillbirth and neonatal death, especially in younger adolescent pregnancies.

## Background

Adolescent pregnancy is defined as pregnancy of teenagers at the age of 10–19 years [1]. About 11% of newborns worldwide are delivered by adolescent women, the pregnancy complications of adolescent women account for 23% of women of all ages, and more than 90% of them occur in developing countries [2]. Compared with the pregnancy of adult women, the pregnancy of adolescent women usually increases the risk of adverse pregnancy outcomes, including fetal growth restriction, preterm delivery and neonatal death [3]. The increased risk of pregnancy of adolescent women is due to their physiological and psychological immaturity and insufficient sexual and reproductive knowledge [4, 5].

The adverse pregnancy outcomes caused by teenage pregnancy are major public health problems with significant social impact [6, 7]. Adolescent pregnancy is a serious problem in the world, pregnancy complications are the major cause of death in adolescent women [2, 8]. Although the rate of adolescent pregnancy has declined, with the increasing of adolescent population, the number of adolescent pregnancy is large, especially in developing countries [9, 10].

The World Health Organization (WHO) reported that the global adolescent pregnancy rate was 10.3% in 2014, the highest rate was 28.8% in Nicaragua, whereas the lowest rate was 0.7% in Japan, an economically developed country [2]. In Bangladesh, with a low economic level in South Asia, the adolescent pregnancy rate in 2015 was 20.2% [11]. In Africa, adolescent pregnancy rate was 18.8% from 1990 to 2018 [6], and even more than 50% in sub-Saharan areas, with 44% of its population below 15 years old [12]. In rural Cameroon, the adolescent pregnancy rate was 20.4% from 2009 to 2016 [13]. In Ethiopia, Africa’s second most populous country, the adolescent pregnancy rate was 13% in 2016 [14]. In UK, the adolescent pregnancy rate was 19.4% [15] from 1950 to 2010. In Romania, the adolescent pregnancy rate was 29.1% from 2007 to 2014 [16]. In Australia, the adolescent pregnancy was 13.1% in 2016 [17].

China is the most populous country in the world. According to China’s 2010 census data [18], 8.5% of women aged 10–50 years were adolescent women, but there were few studies on the topic of adolescent pregnancy. Hebei Province is a province in China with plateaus, mountains, hills, plains, lakes and seashores, its economic level is in the middle level in China. The population of Hebei Province was 75,195,200 according to China’s 2010 census data [18]. This study analyzed the delivery data of 22 hospitals from Hebei Province Maternal Near Miss Surveillance System (HBMNMSS) from 2013 to 2017, and evaluated the adverse pregnancy outcomes of adolescent women.

## Methods

### Study population

HBMNMSS is the sub-database of China’s National Maternal Near Miss Surveillance System (NMNMSS), 22 hospitals (tertiary, secondary, primary hospitals) were enrolled from Hebei Province on the basis of random stratified cluster sampling, with 289,895 registered pregnant women who gave birth during the period from January 1, 2013 to December 31, 2017. We had a permission to access the database of HBMNMSS with Hebei Women and Children’s Health Center. Detailed sampling strategy was described in the previous paper [19].

From this database, data was collected in each hospital using a specially designed data collection form by trained doctors, inputted and sorted out by special personnel. This form included sociodemographic characteristics, obstetric history, place and mode of delivery, pregnancy outcomes, complications during pregnancy, delivery, and in the postpartum period.

Adolescent pregnant women (aged 10–19 years) were 4125 (1.4%), adult pregnant women (aged 20–34 years) were 250,522 (86.4%), pregnant women of advanced maternal age (≥35 years old) were 27,140 (9.4%), and pregnant women with missing data of age were 8108 (2.8%). We enrolled 238,598 singleton pregnant women aged 10–34 years, who were divided into two groups, adolescent group (aged 10–19 years) and adult group (aged 20–34 years). The adolescent group was divided into two subgroups: younger adolescent group (10–17 years) and older adolescent group (18–19 years), and adult group was divided into two subgroups: younger adult group (20–24 years) and older adult group (25–34 years).

### Adverse maternal and perinatal outcomes

The maternal and perinatal outcomes in our study pertained to the latest pregnancy. The adverse maternal outcomes were defined as: cesarean section delivery (CS), anemia, gestational diabetes mellitus (GDM), pre-eclampsia (PE), HELLP syndrome (hemolysis, elevated liver enzymes, and low platelet count syndrome), placenta previa, placental abruption, postpartum hemorrhage, maternal death. The adverse perinatal outcomes were defined as: small for gestational age (SGA), preterm delivery, neonatal low Apgar score, stillbirth, and neonatal death. The CS included emergency CS and elective CS. Anemia was defined as hemoglobin value below 110 g/L. GDM was diagnosed that fasting ≥5.1 mmol/L, one-hour post-prandial ≥10.0 mmol/L, two-hour post-prandial ≥8.5 mmol/L in 75 g oral glucose tolerance test [20]. Pre-eclampsia was defined as hypertension after 20 weeks of gestation (systolic blood pressure ≥ 140 mmHg or diastolic blood pressure ≥ 90 mmHg), with proteinuria (≥300 mg/day) or liver, kidney functional failure, or nerve, blood system abnormalities [21]. HELLP syndrome was a serious complication of pre-eclampsia, including hemolysis, elevated liver enzymes, thrombocytopenia [21]. Hemorrhage was defined as blood loss ≥1000 ml. SGA was the tenth percentile of fetal birth weight below the gestational age [22]. Preterm delivery was defined as delivery between 28 and 36^+ 6^ gestational weeks [23]; Low Apgar score was ≤7 at 5 min. Stillbirth was defined as intrauterine fetal demise at ≥28 weeks’ gestation with the fetus weighing 1000 g or above. Neonatal death included those died at birth, or within 7 days of delivery.

### Statistical analysis

First, we compared the basic data between the two groups of maternal age using T-test or Chi-square test. The counting data were described as n (%) using Chi-square test, and the measurement data were described as mean ± standard deviation (mean ± SD) using T-test. Next, we compared the incidence of adverse pregnancy outcomes between the adolescent group and adult group by Chi-square (휒^2^) test. Two groups were compared using univariate and multivariate logistic regression to evaluate the effect of adolescent group on the risk of adverse pregnancy outcomes, considering the adult group as the reference group. We made the subgroup analysis using logistic regression. The structural equation model mapped out the correlation to each other. Multivariate logistic regression was used to adjust the confounding factors such as: maternal education time, marital status, place of residence, delivery place, parity, history of cesarean delivery. The result was presented as risk ratio (RR) or adjusted risk ratio (aRR) and 95% confidence interval (CI). *P* < 0.05 was considered statistically significant, and all statistical tests were two-tailed. All data were analyzed using SPSS Version 17.0 (RRID: SCR-002865). Stata Version 15.0 (RRID: SCR-012763) was used to generate forest plots.

## Results

**Fig. 1:**
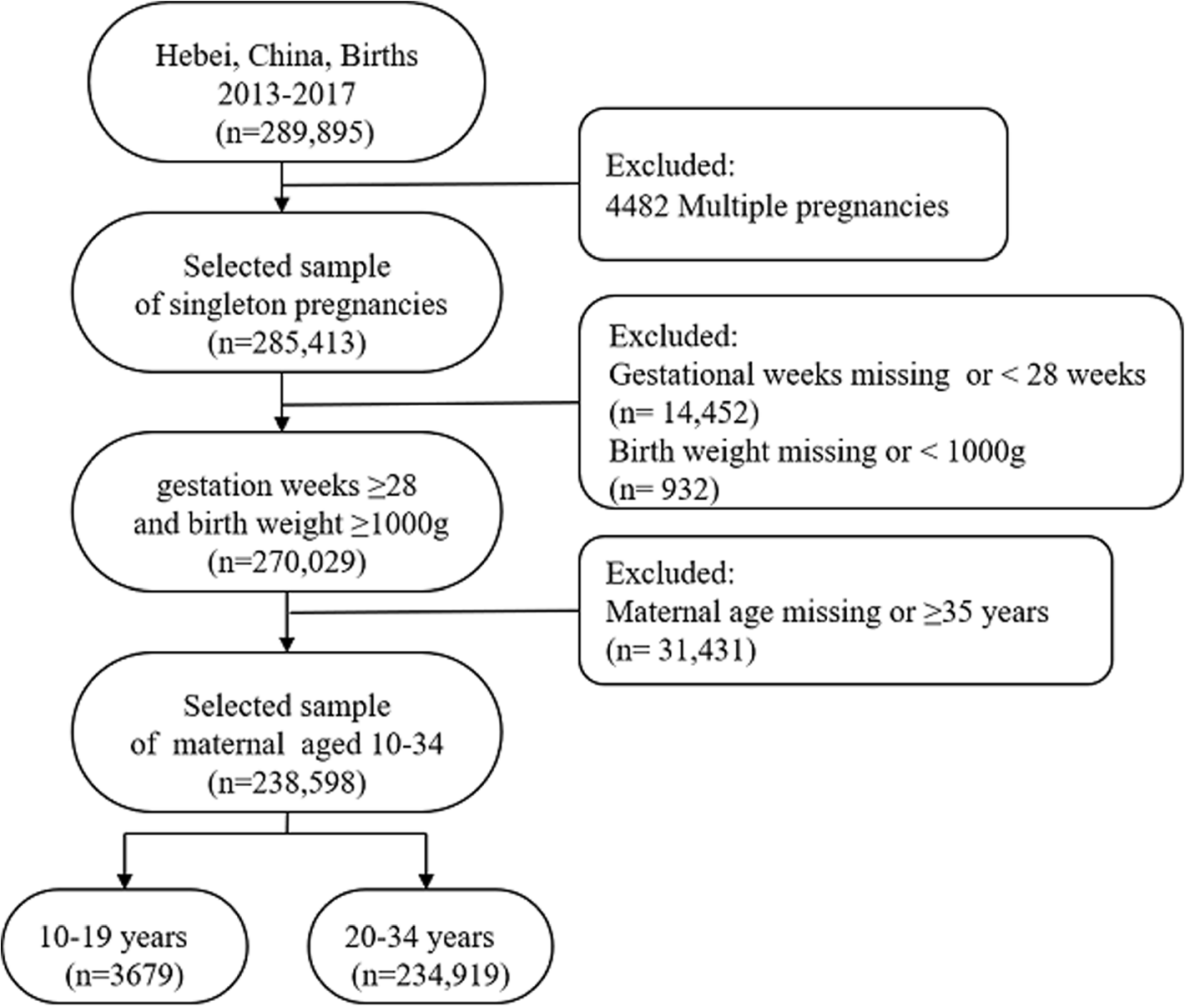
The Flow Chart of Case Selection from 2013 to 2017, Hebei Province, China. Data of the pregnant women was collected from Hebei Province Maternal Near Miss Surveillance System (HBMNMSS)

**Fig. 2:**
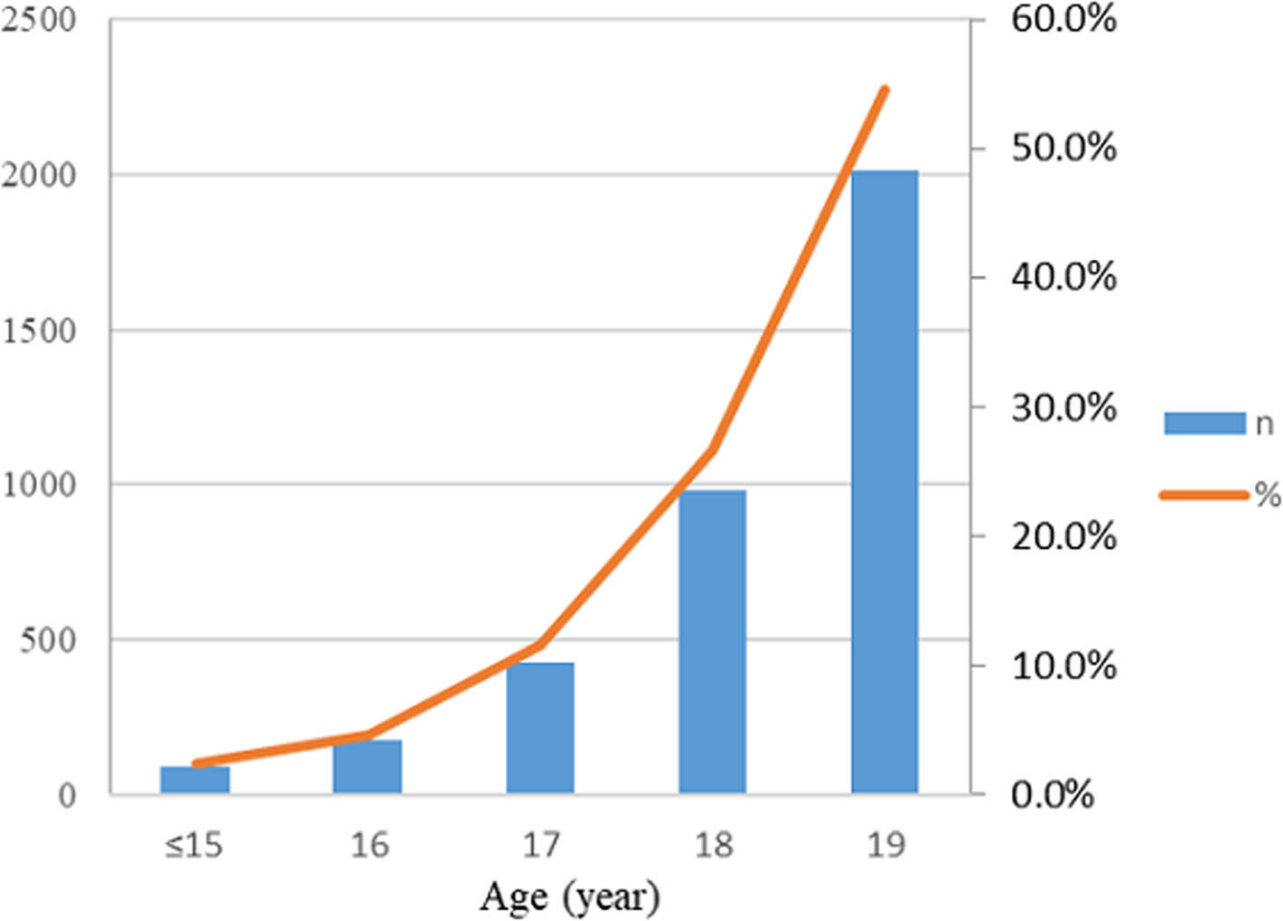
Number and Percentage of Adolescent Pregnant Women in Hebei Province, China, from 2013 to 2017. Bar chart represents the number and line chart represents the percentage. Among the adolescent pregnant women, pregnant women ≤15 years was 88 (2.4%), 16 years was 174 (4.7%), 17 years was 428 (11.6%), 18 years was 979 (26.6%), 19 years was 2010 (54.6%)

The data of 289,985 pregnant women were collected from HBMNMSS, covering the period from 2013 to 2017; excluding multiple pregnancies (*n* = 4482), gestational weeks missing or < 28 weeks (*n* = 14,452), birth weight missing or < 1000 g (*n* = 932), maternal age missing or ≥ 35 years old (*n* = 31,431). Our study analyzed 238,598 singleton pregnant women aged 10–34 years. These pregnant women were divided into two groups, the adolescent group (aged 10–19 years, *n* = 3679) and the adult group (aged 20–34 years, *n* = 234,919). The flow chart of case selection was shown in Fig. 1. In the adolescent group, pregnant women ≤15 years old was 88 (2.4%), 16 years old was 174 (4.7%), 17 years old was 428 (11.6%), 18 years old was 979 (26.6%), 19 years old was 2010 (54.6%) (Fig. 2).

In terms of marital status and education, more adolescent pregnant women were single than adult pregnant women (5.1% versus 0.4%) (*P* < 0.001), meanwhile adolescent pregnant women had lower education than adult pregnant women, with education period ≤6 years (6.1% versus 1.8%) and 7–9 years (62.2% versus 34.3%) (*P* < 0.001). The proportion of adolescent pregnant women that made delivery in secondary hospitals and at home was more than that of the adult pregnant women (*P* < 0.001). Number of prenatal cares in the adolescent group (5.6 ± 2.2) was less than that in the adult group (6.8 ± 2.4) (*P* < 0.001). Birth weight of adolescent pregnant women was lower than that of the adult pregnant women (3204 ± 509 g versus 3344 ± 491 g) (P < 0.001) (Table 1). Compared with women aged 20–24 years and 25–34 years, women aged 10–19 years had lower rate of cesarean delivery and GDM (P < 0.001), but had higher rate of PE, HELLP syndrome, placenta previa, maternal death, preterm delivery, SGA, stillbirth, neonatal death (*P* < 0.05) (Table 2).

**Table 1:** Sociodemographic and Obstetric Characteristics of 238,598 Pregnant Women in Hebei Province, China

	10–19 years (*n* = 3679)	20–34 years (*n* = 234,919)	F/χ2	*P*
Marital status				
Married	3492 (94.9)	234,033 (99.6)		
Single	187 (5.1)	886 (0.4)	1791.693	0.000
Education (year)				
≤ 6	222 (6.1)	4258 (1.8)		
7–9	2265 (62.2)	79,550 (34.3)		
10–12	899 (24.7)	70,870 (30.5)		
> 12	253 (7.0)	77,343 (33.3)	1946.923	0.000
Delivery place				
Tertiary Hospital	950 (25.8)	108,383 (46.1)		
Secondary Hospital	2722 (74.0)	126,348 (53.8)		
Primary Hospital	0 (0.0)	19 (0.0)		
Home	7 (0.2)	163 (0.1)	607.250	0.000
Prenatal care	5.6 (2.2)	6.8 (2.4)	889.906	0.000
Parity				
0	3328 (90.5)	127,414 (54.3)		
1	315 (8.6)	98,078 (41.8)		
≥ 2	35 (1.0)	9324 (4.0)	1918.563	0.000
History of miscarriage	421 (11.4)	59,531 (25.4)	372.160	0.000
Chronic hypertension	15 (0.4)	1352 (0.6)	1.790	0.181
Gestational age (weeks)	38.9 (1.9)	38.9 (1.5)	0.000	0.993
Birth weight (g)	3204 (509)	3344 (491)	292.171	0.000

Data of the pregnant women was collected from Hebei Province Maternal Near Miss Surveillance System (HBMNMSS) from 2013 to 2017. The values shown are n (%), Birth weight shown is mean (SD)

**Table 2:** Adverse Maternal and Perinatal Outcomes of Pregnant Women in HBMNMSS from 2013 to 2017

Outcomes	10–19 years	20–24 years	25–34 years	F/χ2	*P*
	(*n* = 3679)	(*n* = 53,383)	(*n* = 181,536)		
	*n* (%)	*n* (%)	*n* (%)		
Cesarean delivery	1439 (39.1)	24,228 (45.4)	94,563 (52.1)	931.997	0.000
Anemia	900 (24.5)	12,683 (23.8)	48, 367 (26.6)	182.959	0.000
Gestational diabetes mellitus	49 (1.3)	1035 (1.9)	7371 (4.1)	596.689	0.000
Preeclampsia	95 (2.6)	1360 (2.5)	4287 (2.4)	6.575	0.037
HELLP syndrome	5 (0.1)	25 (0.0)	144 (0.1)	8.01	0.018
Placenta previa	7 (0.2)	99 (0.2)	825 (0.5)	80.645	0.000
Placental abruption	11 (0.3)	129 (0.2)	477 (0.3)	0.949	0.622
Postpartum hemorrhage	11 (0.3)	135 (0.3)	565 (0.3)	4.726	0.094
Maternal death	1 (0.0)	4 (0.0)	3 (0.0)	10.525	0.005
Preterm delivery	275 (7.5)	2752 (5.2)	9504 (5.2)	37.638	0.000
Small for gestational age	528 (14.4)	6507 (12.2)	20,977 (11.6)	40.595	0.000
Lower Apgar at 5 min	12 (0.3)	136 (0.3)	388 (0.2)	4.953	0.084
Stillbirth	41 (1.1)	221 (0.4)	593 (0.3)	68.637	0.000
Neonatal death	18 (0.5)	121 (0.2)	332 (0.2)	20.49	0.000

Data of the pregnant women was collected from Hebei Province Maternal Near Miss Surveillance System (HBMNMSS) from 2013 to 2017. The values shown are n (%). HELLP syndrome: hemolysis, elevated liver enzymes, and low platelet count syndrome

**Fig. 3:**
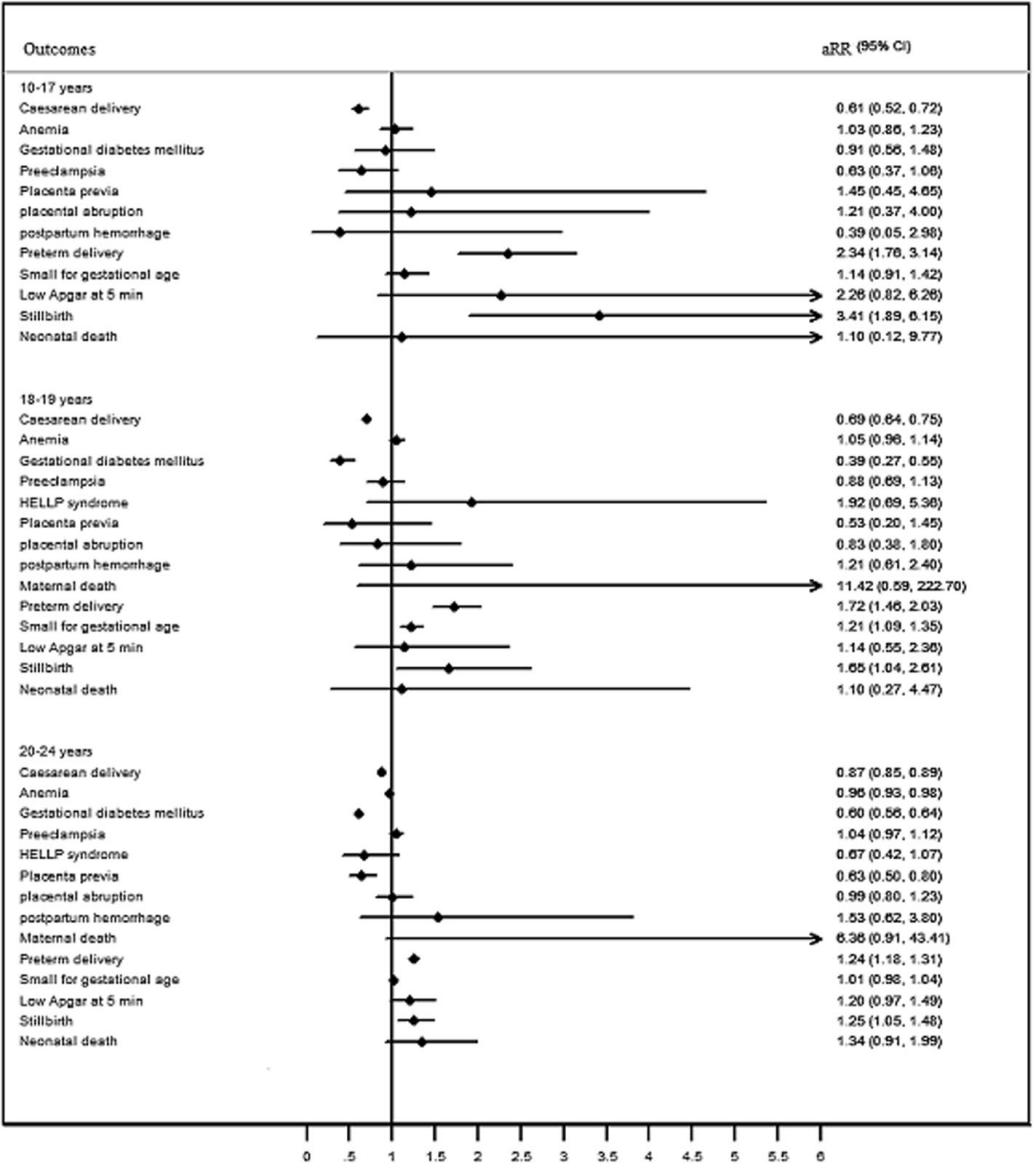
Forest Plots of Risks of Adverse Outcomes of Adolescent Pregnancy Compared with Adult Pregnancy. Compared with women (aged 25–34 years. Adjusted by maternal education, marital status, delivery place, history of miscarriage, parity, previous cesarean delivery

**Fig. 4:**
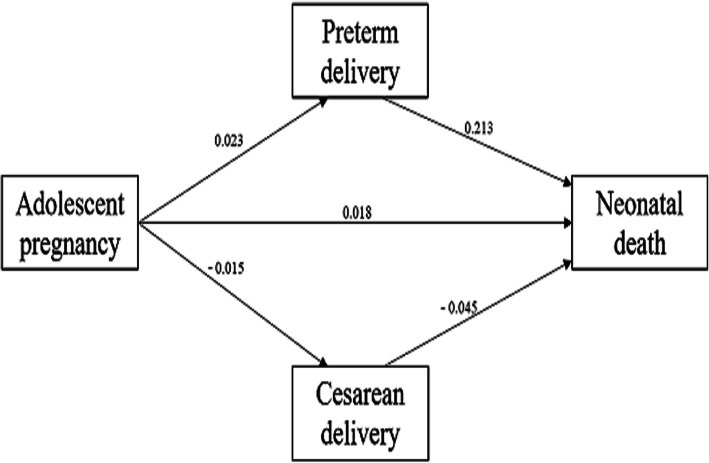
The Structural Equation Model of Risk Factors between Adolescent Pregnancy and Neonatal Death. The path coefficients was showed in figure

Compared with women aged 20–34 years, women aged 10–19 years had a significant lower risk of cesarean delivery (aRR: 0.75, 95%CI: 0.70–0.80), GDM (aRR: 0.55, 95%CI: 0.41–0.73). And adolescent pregnancy had a significantly higher risk of preterm delivery (aRR: 1.76, 95%CI: 1.54–2.01), SGA (aRR: 1.19, 95%CI: 1.08–1.30), stillbirth (aRR: 2.58, 95%CI: 1.83–3.62), neonatal death (aRR: 2.63, 95%CI: 1.60–4.32). There had lower risk of anemia (RR: 0.92, 95%CI: 0.86–0.99) in adolescent group, and higher risk of maternal death (RR: 9.12, 95%CI: 1.12–74.18), but there were no statistically significant difference in the effects of anemia (aRR: 1.04, 95%CI: 0.96–1.12) and maternal death (aRR: 2.61, 95%CI: 0.20–33.77) after adjusting the confounding factors. There was no difference in the risk of, PE, HELLP syndrome, placenta previa, placental abruption, and low Apgar at 5 min in 10–19 years old adolescent pregnancies. Compared with adult group (aged 25–34 years), there were some differences in the subgroup of younger adolescents (aged 10–17 years) and older adolescents (aged 18–19 years) in the risks of adverse pregnancy outcomes. There was no statistical difference between the pregnant women aged 10–17 years and adult group in terms of GDM (aRR: 0.87, 95%CI: 0.54–1.44), and SGA (aRR: 1.11, 95%CI: 0.89–1.39). However, the pregnant women aged 10–17 years had significantly higher risk of stillbirth (aRR: 5.69, 95%CI: 3.36–9.65) and neonatal death (aRR: 7.57, 95%CI: 3.74–15.33). The pregnant women aged 18–19 years had a significantly higher risk of HELLP syndrome (aRR: 2.91, 95%CI: 1.14–7.40). Younger adults (20–24 years) had higher risks of preterm delivery (aRR: 1.26, 95%CI: 1.20–1.32), stillbirth (aRR: 1.45, 95%CI: 1.23–1.72), and neonatal death (aRR: 1.51, 95%CI: 1.21–1.90) than women aged 25–34 years (Table 3 and Fig. 3). The structural equation model showed that the direct effect of adolescent pregnancy on neonatal death was 0.018, the indirect effect of adolescent pregnancy on neonatal death via preterm and cesarean delivery was 0.005 and 0.001 respectively, the total effect was 0.024. Preterm and cesarean delivery were intermediate variables between adolescent pregnancy and neonatal death, as shown in Fig. 4 (R^2^ = 0.33). Compared with women aged 20–34 years, nulliparous women aged 10–19 years had higher risks of anemia, preterm delivery, SGA, stillbirth and neonatal death (Table 4).

**Table 3:** Risks of Adverse Outcomes of Pregnant Women

Outcomes	Maternal age (years)	25–34 years old	
		RR (95%CI)	aRR (95%CI)
Cesarean delivery	10–17	0.54 (0.46–0.63)^***^	0.61 (0.52–0.71)^***^
	18–19	0.60 (0.56–0.65)^***^	0.70 (0.65–0.76)^***^
	20–24	0.76 (0.75–0.78)^***^	0.87 (0.85–0.89)^***^
Anemia	10–17	0.85 (0.71–1.02)	1.01 (0.84–1.23)
	18–19	0.90 (0.83–0.98)^*^	1.03 (0.95–1.12)
	20–24	0.86 (0.84–0.88)^***^	0.95 (0.93–0.97)^***^
Gestational diabetes mellitus	10–17	0.60 (0.37–0.97)^*^	0.87 (0.54–1.44)
	18–19	0.26 (0.18–0.36)^***^	0.39 (0.28–0.56)^***^
	20–24	0.47 (0.44–0.50)^***^	0.60 (0.56–0.64)^***^
Pre-eclampsia	10–17	0.98 (0.60–1.61)	0.85 (0.51–1.40)
	18–19	1.12 (0.90–1.41)	1.09 (0.87–1.38)
	20–24	1.08 (1.02–1.15)^*^	1.11 (1.03–1.18)^**^
HELLP syndrome	10–17	–	–
	18–19	2.11 (0.87–5.15)	2.91 (1.14–7.40)^*^
	20–24	0.59 (0.39–0.90)^*^	0.84 (0.54–1.32)
Placenta previa	10–17	0.96 (0.31–2.98)	2.01 (0.64–6.38)
	18–19	0.29 (0.11–0.78)^*^	0.73 (0.27–1.97)
	20–24	0.41 (0.33–0.50)^***^	0.71 (0.57–0.90)^**^
Placental abruption	10–17	1.66 (0.53–5.17)	1.76 (0.55–5.60)
	18–19	1.02 (0.51–2.05)	1.30 (0.64–2.66)
	20–24	0.92 (0.76–1.12)	1.15 (0.93–1.42)
Postpartum hemorrhage	10–17	0.47 (0.07–3.31)	0.55 (0.08–3.96)
	18–19	1.08 (0.58–2.01)	1.49 (0.79–2.83)
	20–24	0.81 (0.67–0.98)^*^	1.03 (0.84–1.26)
Maternal death	10–17	–	–
	18–19	20.25 (2.11–194.74)^**^	11.02 (0.72–169.77)
	20–24	4.53 (1.02–20.26)^*^	5.01 (0.91–27.40)
Preterm delivery	10–17	2.08 (1.62–2.66)^***^	2.57 (1.98–3.34)^***^
	18–19	1.33 (1.15–1.53)^***^	1.77 (1.51–2.06)^***^
	20–24	0.98 (0.94–1.03)	1.26 (1.20–1.32)^***^
Small for gestational age	10–17	1.22 (0.98–1.52)	1.11 (0.89–1.39)
	18–19	1.30 (1.17–1.44)^***^	1.20 (1.08–1.33)^***^
	20–24	1.06 (1.03–1.09)^***^	1.00 (0.97–1.03)
Low Apgar at 5 min	10–17	2.82 (1.05–7.58)^*^	2.87 (1.05–7.87)^*^
	18–19	1.26 (0.63–2.54)	1.39 (0.68–2.83)
	20–24	1.19 (0.98–1.45)	1.31 (1.06–1.62)^*^
Stillbirth	10–17	8.17 (5.08–13.14)^***^	5.69 (3.36–9.65)^***^
	18–19	2.37 (1.56–3.59)^***^	2.33 (1.50–3.60)^***^
	20–24	1.27 (1.09–1.48)^***^	1.45 (1.23–1.72)^***^
Neonatal death	10–17	7.39 (3.79–14.38)^***^	7.57 (3.74–15.33)^***^
	18–19	1.66 (0.85–3.21)	2.03 (1.03–4.00)^*^
	20–24	1.24 (1.01–1.53)^*^	1.51 (1.21–1.90)^***^

The pregnant women aged 25–34 years as the reference group. Adjusted by maternal education, marital status, delivery place, history of miscarriage, parity, previous cesarean delivery. *P < 0.05, **P < 0.01, ***P < 0.001

**Table 4:** Risk of Adverse Pregnancy Outcomes in Adolescent Pregnancy Stratified by Parity

Outcomes	Nulliparous (*n* = 3328)		Multiparous (*n* = 350)	
	RR (95%CI)	aRR (95%CI)	RR (95%CI)	aRR (95%CI)
Cesarean delivery	0.73 (0.68–0.78)^***^	0.69 (0.65–0.75)^***^	0.55 (0.44–0.68)^***^	0.76 (0.57–1.02)
Anemia	1.08 (1.00–1.17)	1.10 (1.02–1.20)^*^	0.80 (0.63–1.02)	0.86 (0.67–1.10)
Gestational diabetes mellitus	0.36 (0.27–0.48)^***^	0.59 (0.44–0.79)^***^	0.25 (0.08–0.77)^*^	0.37 (0.12–1.16)
Pre-eclampsia	1.08 (0.87–1.33)	0.98 (0.79–1.21)	0.39 (0.12–1.20)^*^	0.46 (0.15–1.43)
HELLP syndrome	2.00 (0.66–6.06)	2.58 (0.96–6.90)	–	–
Placenta previa	0.50 (0.19–1.35)	0.76 (0.28–1.07)	1.49 (0.48–4.66)	2.60 (0.81–8.33)
Placental abruption	1.41 (0.77–2.57)	1.44 (0.77–2.69)	–	–
Postpartum hemorrhage	1.10 (0.61–2.01)	1.39 (0.75–2.58)	–	–
Maternal death	9.57 (1.07–85.68)^*^	2.14 (0.14–31.82)	–	–
Preterm delivery	1.56 (1.37–1.77)^***^	1.67 (1.45–1.92)^***^	1.16 (0.75–1.79)	1.34 (0.84–2.14)
Small for gestational age	1.27 (1.15–1.39)^***^	1.22 (1.11–1.35)^***^	0.78 (0.54–1.12)	0.72 (0.49–1.05)
Low Apgar at 5 min	1.75 (0.98–3.12)	1.57 (0.86–2.86)	–	–
Stillbirth	3.83 (2.74–5.36)^***^	2.55 (1.76–3.71)^***^	2.15 (0.69–6.71)	2.00 (0.64–6.31)
Neonatal death	2.80 (1.71–4.59)^***^	2.72 (1.61–4.61)^***^	1.42 (0.20–10.12)	1.57 (0.22–11.26)

Adjusted by maternal education, marital status, delivery place, history of miscarriage, previous cesarean delivery. Women aged 20–34 years as the reference group. *P < 0.05, **P < 0.01, ***P < 0.001

## Discussion

The rate of adolescent pregnancy (aged 10–19 years) was 1.4% in Hebei Province, China from 2013 to 2017, lower than 4.3% from 2010 to 2011 reported by Ganchimeg, T. et al. [2]. The difference of the rate may result from the difference of areas, periods and data-collection system. We collected the data of 289,859 pregnant women in Hebei Province for 5 years, which is a developed area in eastern China. Ganchimeg T. et al. [2] collected the data of 314,623 pregnant women from 29 countries for 2 to 4 months, but their sample size from China was smaller than ours. Ganchimeg T. et al. [2] collected the data only from the tertiary hospitals, but our data was collected from all levels of hospitals (including tertiary hospitals, secondary hospitals, primary hospitals). Therefore, our research was representative of the situation of adolescent pregnancy in Hebei Province of China.

Consistent with several previous studies worldwide [1, 2, 13, 14, 24–26], our study showed that, compared with the adult pregnant women (aged 20–34 years), the adolescent pregnant women had significantly lower level of education, less numbers of prenatal care, and higher single-hood rate. Our data showed that there were more adolescent pregnant women that delivered in provincial and municipal hospitals than adult pregnant women, and those adolescent women that delivered at home were 2–3 times more of the adult group. Moreover, the number of unmarried adolescent pregnant women was 10 times higher than that in adults. The reasons for the situation above could be attributed to the factors such as poverty, low education, lack of sex education and contraceptive methods [12, 27]. In addition, adolescent pregnant women lacked economic independence [11], and they might be difficult to deal with the burden of pregnancy [13].

The risk of cesarean delivery in adolescent pregnancy reduced by 25% than adult pregnancy, which was consistent with the previous found, with aRR (95%CI) varying from 0.49 (0.42–0.59) to 0.79 (0.75–0.89) [1, 2, 13, 16, 28, 29]. In fact, adolescent women were at the stage of physical growth, with immature reproductive system, and the incidence of cephalopelvic disproportion in adolescent pregnancy was higher than that of adult women [30]. But why was the rate of cesarean delivery in adolescent pregnancy was lower than that of the adult pregnancy? First, the adolescent women aged 18–19 years accounted for more than half of all adolescent pregnant women, their physical development was basically mature [31]. Second, the pelvic cavity of adolescent women was smaller than that of adult women, and the fetal weight was relatively lower [32]. More preterm delivery and lower fetal weight was conducive for the adolescent women to make vaginal delivery [2, 33]. And for the younger adolescent women (aged 10–17 years), the risk of cesarean delivery even could reduce by 49%, which might be related to their limited access to cesarean delivery [2].

The endocrine regulating system of adolescents is functions differently from that of adults or is not fully developed [34], with higher risks of obesity or GDM, but we found a lower risk of GDM in older adolescents (18–19 years) and younger adults (20–24 years) compared with women aged 25–34 years. This result was basically consistent with another study on the delivery population in Beijing, China, with aRR (95%CI) was 0.55 (0.39–0.77) [28]. Marvin-Dowle, K. et al. [29] showed that the risk of GDM in adolescent pregnancy of Pakistani and white British was similar to our results, with aRR (95%CI) value of 0.35 (0.20–0.62), but the risk value was lower, which may be caused by different races. Further studies would be made to investigate the reasons of the lower risk of GDM in adolescents.

We found that the risk of stillbirth and neonatal death in adolescent pregnancy aged 10 to 19 years was 2.58 times and 2.63 times of that in adults pregnant women, in the younger pregnant women aged 10 to 17 years, the risk was higher (stillbirth, aRR: 5.69, 95%CI: 3.36–9.65 and neonatal death, aRR: 7.57, 95%CI: 3.74–15.33). We got the similar results on stillbirth as Ganchimeg, T. et al. [30], with aRR (95%CI): 1.32 (1.11–1.57) for the younger adolescent pregnant women aged 16 to 17 years, which also was in line on perinatal death as the report of Althabe, F [25]., with aRR (95%CI): 1.13 (1.02–1.25) for the adolescent pregnant women aged 15 to 19 years. Our study confirmed the increased risk of preterm delivery (aRR: 1.76, 95%CI:1.54–2.01) in adolescent than adults. Our study got the corroborated results as many other studies on the risk of preterm delivery in adolescence, with aRR (95%CI) varying from 1.18 (1.11–1.27) to 2.15 (1.26–3.67) [1, 2, 24, 28, 29]. On the contrary, Althabe, F.et al. [25] found that African-American adolescent women had lower preterm delivery rate than adult women, which might be due to ethnic difference. The increased risk of SGA [aRR (95%CI): 1.19 (1.08–1.30)] in adolescent pregnancy in our study was consistent with that of Agbor, V. N. et al. [13], with aRR (95%CI): 1.7(1.1–2.6). Younger adults (20–24 years) had higher risks of preterm delivery, stillbirth, and neonatal death than adult women aged 25–34 years, but lower than adolescent women. Adolescent women were at the stage of development, their uteri were immature, the blood supply to the placenta was affected by the competition between mother and fetus. Preterm delivery and SGA were associated with maternal malnutrition [35], and preterm delivery was positively associated with the risk of perinatal death [36]. Appropriate prenatal care could reduce the incidence of preterm delivery and stillbirth [37]. With less perinatal care and insufficient nutrition during pregnancy, the intrauterine growth and development of the fetus was affected. And the less perinatal care the adolescent pregnant women had during their pregnancies, the less preventive intervention on time they would take [38]. We believed that with the maturity of reproductive system, the incidence of adverse outcomes were gradually decreased.

The adverse maternal outcomes of the adolescent pregnant had no difference from those of adult pregnant in our study, such as in PE, placenta previa, placental abruption, postpartum hemorrhage, and some studies were consistent with our findings [13, 16, 25, 26, 29]. Some studies found that adolescent pregnancy increased the risk of postpartum hemorrhage [1, 16] and PE [1]. Other studies found that adolescent pregnancy reduced the risk of postpartum hemorrhage [24] and PE [2]. We think that race, local medical skill level and sample size may be related to these differences. Previous report has found that adolescent pregnancy was a risk factor for increased maternal mortality [39, 40]. We found that maternal mortality in adolescent pregnancy was higher than that of adult women. However, adolescent pregnancy was not a risk factor for maternal mortality after adjusting the confounding factors. The increase in maternal mortality during adolescent pregnancy was related to the social factors such as maternal poverty and education [40], and the lack of medical resources may also affect maternal mortality [41]. After structural equation model analysis, we also found that preterm delivery and cesarean delivery had an indirect effect on the correlation between adolescent pregnancy and neonatal death. The total effect of adolescent pregnancy on neonatal death increased via the indirect effects of preterm and cesarean delivery.

We used the government-funded HBMNMSS as our data source, the data covered 10 cities, with large sample size and representative population. The data was collected not only from tertiary hospitals, but also from secondary and primary hospitals, which could truly reflect the situation of adolescent pregnancy in Hebei Province. But there were some limitations in our research, vast majority of adolescent pregnant women were 18–19 years old, the conclusion was mainly based on the older adolescent pregnant women. The database did not contain the information about smoking and maternal BMI and economic status. These confounding factors might affect the adverse pregnancy outcomes. The diagnosis system was not widely used in Hebei Province, which might lead to some bias in our study.

## Conclusion

The adolescent pregnancy was related to adverse perinatal (fetal and neonatal) outcomes, such as preterm birth, stillbirth and neonatal death, especially in younger adolescent pregnancies, but it was a protective factor for cesarean delivery and GDM. Adolescence was not the best period for pregnancy. Health education and health care should be strengthened to improve the outcomes of adolescent pregnancy.

## Data Availability

The datasets used and/or analysed during the current study are available from the corresponding author on reasonable request.

## Abbreviations

GDM: Gestational diabetes mellitus
SGA: Small for gestational age
PE: Preeclampsia
HELLP syndrome: Hemolysis, elevated liver enzymes, and low platelet count syndrome
HBMNMSS: Hebei province Maternal Near Miss Surveillance System
